# A novel outpatient desensitization protocol for recombinant human erythropoietin allergy in a pediatric patient

**DOI:** 10.1186/s13223-018-0233-1

**Published:** 2018-03-12

**Authors:** Jaime S. Rosa, Van B. Vuong, Orly Haskin, Anne Y. Liu

**Affiliations:** 1 Division of Allergy, Immunology, and Rheumatology, Department of Pediatrics, Stanford University School of Medicine, 269 Campus Drive, CCSR 3215, MC 5366, Stanford, CA 94305 USA; 2 Lucile Packard Children’s Hospital Stanford, Stanford Children’s Health, Palo Alto, CA 94304 USA; 3 Division of Nephrology, Department of Pediatrics, Stanford University School of Medicine, Stanford, CA 94305 USA

**Keywords:** Hypersensitivity, Epoetin alfa, Desensitization, Pediatric, Child, Drug allergy

## Abstract

**Background:**

Recombinant human erythropoietin, such as epoetin alfa and darbepoetin alfa, is an important therapy for anemia due to chronic renal failure. Allergy to recombinant human erythropoietin and the need for desensitization are rare.

**Case presentation:**

We report here a novel epoetin alfa outpatient desensitization protocol in a girl who developed delayed cutaneous hypersensitivity to subcutaneous epoetin alfa and intravenous darbepoetin alfa. An initial attempt at traditional epoetin alfa desensitization failed, so we created a slower 17-day outpatient desensitization that succeeded and allowed treatment continuation.

**Conclusions:**

This case highlights the notion that delayed-type hypersensitivity to recombinant human erythropoietin can occur as evident by reproducible reactions after repeated exposures and slow outpatient desensitization can be considered when a trial of more rapid induction of tolerance is unsuccessful.

## Background

Recombinant human erythropoietin is a mainstay treatment for anemia associated with chronic renal failure. These recombinant products, including epoetin alfa, have amino acid compositions similar to natural human erythropoietin but can still trigger unintended immunologic reactions [1]. Desensitization may be an option to allow continued therapy after development of a drug allergy.

Reports of hives or anaphylaxis immediately following administration of epoetin alfa have suggested IgE-mediated hypersensitivity [1–5]. Some of these patients were able to tolerate epoetin alfa after 3-h or 2-day desensitization [3, 4, 6]. Other reports describe delayed-type hypersensitivity (DTH) rashes [7]. Rarely, acute generalized exanthematous pustulosis can occur weeks after starting epoetin alfa [6]. Slow outpatient desensitization to epoetin alfa has not been described. We present a case of a patient who failed a previously published 2-day epoetin alfa desensitization regimen but tolerated epoetin alfa after desensitization over 17 days.

## Case presentation

**Fig. 1:**
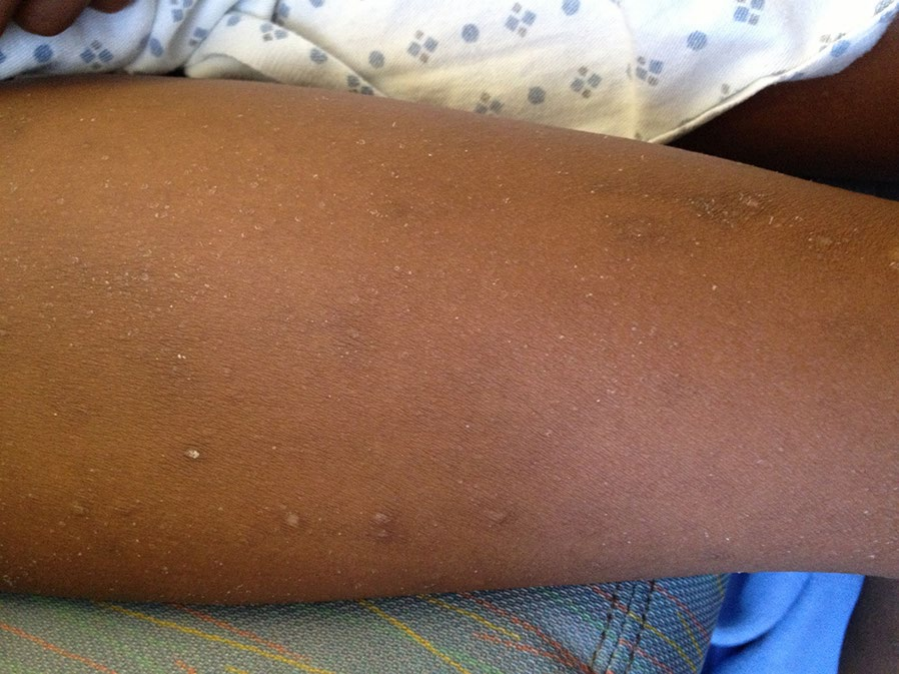
The patient developed an intensely pruritic, maculopapular rash during the second day of the first desensitization protocol

An 11-year-old girl with end stage renal disease secondary to focal segmental glomerulosclerosis was admitted for bilateral nephrectomies and initiation of hemodialysis. 6 weeks prior to hospitalization, the patient was started on subcutaneous epoetin alfa. After three injections, she developed a pruritic rash that gradually improved over 2 weeks. On this admission, she had a hemoglobin concentration of 6.5 g/dL. Epoetin alfa was switched to intravenous darbepoetin alfa due to the reported rash. The following day, she developed a diffuse, pruritic, maculopapular rash (Fig. 1). Eosinophil count was 210 cells/µL, and aspartate and alanine aminotransferases were within the normal ranges. Hydroxyzine alleviated her symptoms, and allergy consultation was requested.

Since the patient required recombinant human erythropoietin to avoid the need for recurrent blood transfusions, we followed a previously published two-day epoetin alfa desensitization protocol reported in an adult who had experienced maculopapular eruptions after receiving epoetin alfa [6]. Our protocol was adjusted for the pediatric patient’s weight to achieve a target dose of 700 IU (Table 1). On day one of desensitization, 7, 14, 28, and 56 IU of epoetin alfa were administered at 6-h intervals. Within 6 h of the 56 IU dose, she developed a pruritic, maculopapular rash, improved with hydroxyzine, and she was started on prednisone 10 mg daily with subsequent rash resolution. The protocol was then modified to lengthen the course, with 48–72 h intervals between each escalating dose, carried out in the outpatient setting (Table 2). After hospital discharge, the patient started the prolonged desensitization, resuming at the previously tolerated dose of 28 IU on day four counting from the first day of initial desensitization. 2 h later, she developed itching in her lower back, thighs, and forearms, but there was no visible lesion. Hydroxyzine 25 mg ameliorated the symptom. On day seven, she received 56 IU of epoetin alfa without further complaints, and thereafter she continued to tolerate escalating doses according to schedule (Table 2). 2 days after achieving the target dose of 700 IU, the prednisone was discontinued. Her eosinophil counts and liver enzymes were monitored at least once weekly during the desensitization without abnormalities (Table 3). The patient continued to tolerate epoetin alfa at 700 IU 3 days a week, which was increased several weeks later to 850 IU 3 days a week. 2 months later, she missed several doses then received intravenous epoetin alfa during peritoneal catheter revision; the next day she developed a papular rash, fever, and eosinophilia, which resolved after several days of low dose prednisone and resumption of her regular subcutaneous dosing. Subsequently, the patient experienced no other adverse effects until this therapy was stopped when she received a deceased-donor renal transplant a year later.

**Table 1:** Original inpatient 2-day slow desensitization protocol

Day	Dose (IU)*	Cumulative dose (IU)
1	7	7
1	14	21
1	28	49
1	56^†^	105
2	112	217
2	224	441
2	259	700

* Doses were administered every 6 h

^†^Desensitization terminated due to reaction

**Table 2:** Modified outpatient 17-day slow desensitization protocol

Day	Dose (IU)*
1	7
1	14
4	28^†^
7	56
10	112
12	224
15	450
17	700

* Doses were administered every 48–72 h

^†^Continued from first trial of desensitization

**Table 3:** Laboratory testing results at different time periods of the patient’s presentation

Laboratory testing	Baseline before recombinant human erythropoietin	Day 2 of initial reaction to intravenous darbepoetin alfa	Day 2 of failed 2-day desensitization to epoetin alfa	During modified desensitization (at 112 IU daily dose)	One month after desensitization
Hemoglobin (g/L)	7.8	7.0	6.7	6.0	9.2
Hematocrit (%)	22.3	20.7	19.5	18.6	27.0
White blood cells (cells/μL)	9100	10,400	13,100	13,300	10,200
Neutrophils (cells/μL)	3600	5700	6100	8900	9200
Lymphocytes (cells/μL)	3600	1300	2800	2400	600
Monocytes (cells/μL)	400	1000	1300	1000	200
Eosinophils (cells/μL)	1400	2400	3000	900	200
Basophils (cells/μL)	100	0	0	100	0
Aspartate aminotransferase (IU/L)	34.0	NM	14.0	15.0	NM
Alanine aminotransferase (IU/L)	33.0	NM	< 15	15.0	NM
Blood urea nitrogen (mg/dL)	51.0	54.0	59.0	9.0	30.0
Creatine (mg/dL)	3.2	8.1	12.9	11.6	7.5

*NM* not measured

## Discussion

Immediate hypersensitivity reactions to human recombinant erythropoietin are characterized by bronchospasm, urticaria, cyanosis, angioedema, vomiting, abdominal discomfort, and hypotension [1–3] occurring within minutes to a few hours of administration. In contrast, our pediatric patient experienced a maculopapular rash 24 h after drug administration. The differential diagnosis for her reaction included IgG-mediated hemolytic anemia (such as pure red cell aplasia) as the patient remained anemic while on recombinant human erythropoietin therapy [5, 8]. However, anti-epoetin alfa and anti-darbepoetin alfa antibodies were non-detectable when biosensor immunoassay was offered and performed by the manufacturer (GE Healthcare-Biocare, Uppsala, Sweden) [9]. Furthermore, she did not have features that would favor drug reaction with eosinophilia and systemic symptoms (DRESS), acute generalized exanthematous pustulosis, Stevens-Johnson syndrome, or other drug eruptions. Although the family declined a skin biopsy for confirmation due to the invasive nature of this procedure, the clinical presentation was consistent with DTH. Therefore, we followed a previously published desensitization protocol [6], which failed to induce tolerance to the medication for our patient, whose rash recurred. Because of the relatively benign nature of her allergic response, we opted to try a novel desensitization protocol, with longer intervals between dose escalations, which can increase the likelihood for successful desensitization [7].

Hypersensitivity to recombinant human erythropoietin and treatment with rapid desensitization have been reported [1, 6, 8], but outpatient desensitization for DTH has not, particularly after failed rapid desensitization. Our protocol features slower dose escalation (17 days), low dose prednisone, and outpatient setting. Whether the corticosteroid contributed to tolerance induction in this case is unknown, but its discontinuation shortly thereafter demonstrated that it was not necessary for maintenance of tolerance.

## Conclusions

Two key points make this case noteworthy. First, we proved an antigen-specific allergy by provoking the same reaction upon re-administration, which is infrequently done in case reports of successful desensitization. Often the severity of reaction makes physicians reluctant to rechallenge. In this case, her reactions from the initial desensitization attempt and subsequent exposure to an intravenous bolus after missing a few subcutaneous doses provided good evidence of a true allergy. Second, the slow desensitization was successful in a patient who had failed rapid desensitization. This bolsters the concept that desensitization protocols must be tailored to the patient’s reaction and any single protocol may not be universally applicable. Outpatient desensitization is a viable option for selected patients who have non-life-threatening DTH to epoetin alfa and require its use, particularly if rapid desensitization is unsuccessful.

## Abbreviations

DTH: delayed-type hypersensitivity
g/dL: gram per deciliter
cells/µL: cells per microliter
IU: international unit
DRESS: drug reaction with eosinophilia and systemic symptoms
